# Timing is everything: Fishing‐season placement may represent the most important angling‐induced evolutionary pressure on Atlantic salmon populations

**DOI:** 10.1002/ece3.3304

**Published:** 2017-08-11

**Authors:** Alison C. Harvey, Yongkai Tang, Vidar Wennevik, Øystein Skaala, Kevin A. Glover

**Affiliations:** ^1^ Institute of Marine Research Bergen Norway; ^2^ Freshwater Fisheries Research Center Chinese Academy of Fishery Sciences Wuxi China; ^3^ Department of Biology Sea Lice Research Centre University of Bergen Bergen Norway

**Keywords:** evolution, fishing, harvest, migration, sex marker, water flow

## Abstract

Fisheries‐induced evolution can change the trajectory of wild fish populations by selectively targeting certain phenotypes. For important fish species like Atlantic salmon, this could have large implications for their conservation and management. Most salmon rivers are managed by specifying an angling season of predetermined length based on population demography, which is typically established from catch statistics. Given the circularity of using catch statistics to estimate demographic parameters, it may be difficult to quantify the selective nature of angling and its evolutionary impact. In the River Etne in Norway, a recently installed trap permits daily sampling of fish entering the river, some of which are subsequently captured by anglers upstream. Here, we used 31 microsatellites to establish an individual DNA profile for salmon entering the trap, and for many of those subsequently captured by anglers. These data permitted us to investigate time of rod capture relative to river entry, potential body size‐selective harvest, and environmental variables associated with river entry. Larger, older fish entered the river earlier than smaller, younger fish of both sexes, and larger, older females were more abundant than males and vice versa. There was good agreement between the sizes of fish harvested by angling, and the size distribution of the population sampled on the trap. These results demonstrate that at least in this river, and with the current timing of the season, the angling catch reflects the population's demographics and there is no evidence of size‐selective harvest. We also demonstrated that the probability of being caught by angling declines quickly after river entry. Collectively, these data indicate that that the timing of the fishing season, in relation to the upstream migration patterns of the different demographics of the population, likely represents the most significant directional evolutionary force imposed by angling.

## 1 INTRODUCTION

Selective harvest through commercial fishing or recreational angling may elicit evolutionary changes in wild fish populations (Hard et al., 2008; Heino & Godoe, 2002), a process known as fisheries‐induced evolution (FIE). Several studies report that long‐term commercial fishing efforts may have altered important life‐history traits of wild fish populations through nonrandom mortality (Dunlop, Enberg, Jørgensen, & Heino, 2009; Kendall, Dieckmann, Heino, Punt, & Quinn, 2013). Similarly, studies show that recreational angling has selectively targeted certain phenotypes in several popular angling species (Alós, Palmer, Linde‐Medina, & Arlinghaus, 2014; Hessenauer et al., 2015). Modeling suggests that shifts in key life‐history traits through fisheries‐induced evolution can take place over relatively small time scales (Barot, Heino, Morgan, & Dieckmann, 2005) and could influence the biological reference points or conservation limits used by fisheries managers to manage wild populations (Heino & Godoe, 2002). Selection targeting less numerous phenotypes, such as larger fish, may have higher evolutionary consequences for freshwater fish like salmonids than for marine stocks with large population sizes, as salmonids tend to have fragmented, low population sizes, and changes to the phenotypic balance of such a population may not be easily reversed (Heino & Godoe, 2002). Fisheries‐induced evolutionary changes may have further implications for population resilience in response to anthropogenic pressures and climate change. Therefore, understanding how selective recreational harvest may influence populations, and potentially affect their evolutionary trajectory, is essential to develop sustainable management approaches.

The Atlantic salmon (*Salmo salar*) is an anadromous salmonid that represents a highly prized social and economic resource throughout its native range. Reproductive and juvenile stages of its life cycle are completed in freshwater streams in the Northern Hemisphere of both sides of the Atlantic, while the marine stage of the life cycle is completed in offshore areas of the Atlantic including the Baltic (Webb, Verspoor, Aubin‐Horth, Romakkaniemi, & Amiro, 2007). The numbers of adult salmon returning to these streams from their ocean feeding grounds are presently, that is, in the period 1970 to 2016, at historically low levels (Forseth et al., 2017; ICES, 2016b). In some regions, populations are in danger of or have already gone extinct (Webb et al., 2007). While the reasons underpinning the reduction in the abundance of adult salmon are diverse and complicated (Parrish, Behnke, Gephard, McCormick, & Reeves, 1998), various anthropogenic factors, including hydroelectric dams, habitat degradation, pollution, climate change, impacts from fish farms, and overexploitation through commercial and recreation fishing have been implicated (ICES, 2016a; Lenders et al., 2016, Parrish et al., 1998).

Most Atlantic salmon enter their natal rivers over a period of several months from late spring to early autumn, although return times vary across their range (Klemetsen et al., 2003). Time of entry to freshwater is linked to various factors, including river characteristics such as temperature and discharge (Jonsson & Jonsson, 2009), and demographic factors such as age and size (Thorstad, Whoriskey, Rikardsen, & Aarestrup, 2011). Differential exploitation of different age/size classes of salmon through angling is inevitable, as the return times of these groups may or may not coincide with an angling season (Borgstrøm et al., 2010; Hard et al., 2008; ICES, 2016a). Examples of shifts in demography toward smaller fish and changes in run times potentially caused by selective angling pressures have been observed in Atlantic salmon populations in Ireland and Spain (Consuegra, Garcia de Leaniz, Serdio, & Verspoor, 2005; Quinn, McGinnity, & Cross, 2006; Saura et al., 2010). Increasing the accuracy of population estimates of salmon stocks is therefore vital for precise monitoring of such evolutionary trends and the management of vulnerable populations.

Salmon populations are typically managed according to biological reference points or conservation limits which refer to harvesting the excess fish while maintaining the spawning requirement in relation to the carrying capacity of the specific river (Forseth et al., 2013; ICES, 2016a). In Norway, salmon rivers are managed with the primary goal of population conservation and the secondary goal of maximizing fishery (primarily angling) yields (Forseth et al., 2013). To provide advice to local river managers about angling season and catch limits based on conservation targets, information on migration timing, spawning biomass and population size of the river needs to be estimated. One of the ways to estimate this is using the nominal angling catch itself, which has inherent bias and may be influenced by unreported catches and release mortality (ICES, 2016a). While diving surveys (drift counts), counting facilities, and tagging experiments supplement these observational methods, these may also have their limitations in providing an unbiased overview of population abundance or migration (Thorstad, Økland, Aarestrup, & Heggberget, 2008). In addition, as stocks decline, angling quotas have been reduced, and thus, catch numbers are low, with further implications for the accuracy of abundance estimates (ICES, 2016a).

**Figure 1:**
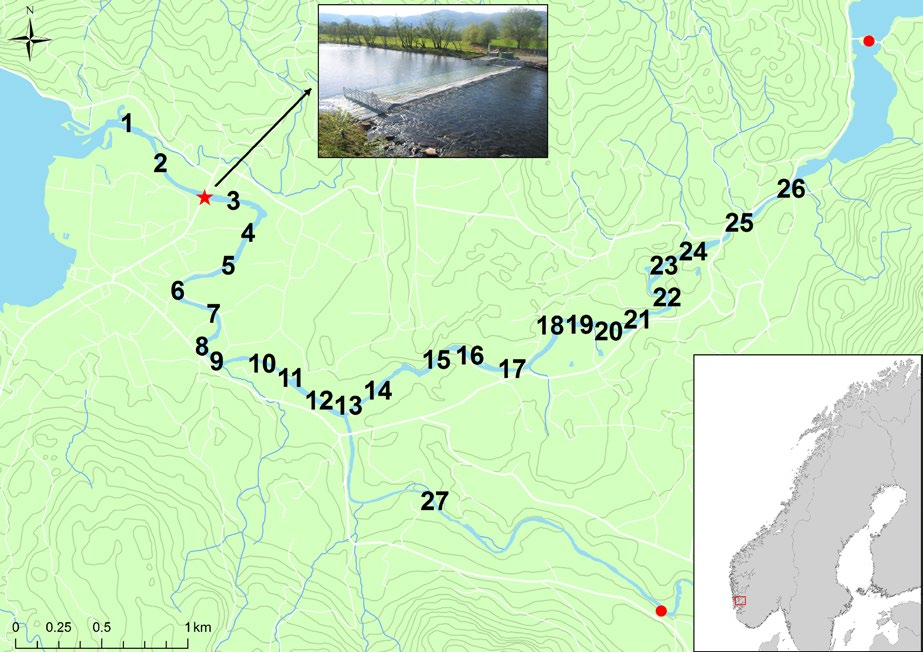
Map of the Etne River showing the fishing zones (numbered 1 to 27), and the two measuring stations for the river discharge (red circles) and the location of the Etne trap in zone 3 (red star). Ascending fish were sampled on the trap between zone 2 and 3 and released back into the river where after some of them were captured by anglers. Some fish were also captured below the trap in fishing zones 1 and 2

The River Etne, located in the Hordaland county of southwest Norway (59°40′N, 5°56′E), supports the largest salmon population in region (annual catch ~3500 kg/year; www.SSB.no (1969–2013)) and has 371,480 m^2^ (Hindar et al., 2007) of river available for juvenile production (Figure 1). This county has the highest density of commercial salmon aquaculture in Norway, and the population inhabiting the river has been significantly admixed due to gene flow from farmed escaped salmon (Glover et al., 2012, 2013; Karlsson, Diserud, Fiske, & Hindar, 2016a). Glover et al. (2013) estimated the level of introgression to be around 20%, while Karlsson, Diserud, Fiske, and Hindar (2016b) estimated the level of introgression in Etne at >10%. In response to this impact, and to prevent further introgression of farmed escaped salmon in this population, a modified Resistance Board Weir (from here on referred to as the trap) was installed near the river mouth (Figure 1). The trap prevents upstream migration for adult salmonids which are channeled into a sluice where they can be sampled and thereafter permitted to continue their upstream migration. Although there are upstream trapping facilities on smaller river systems (e.g., the Burrishoole river system, west Ireland (McGinnity et al., 1997), and the River Bidasoa, north Spain (Saura et al., 2010)), to our knowledge, this is the only such trap installed on the mouth of any major river system in Europe, and therefore provides a unique opportunity to study the adult population migration and harvest patterns through angling in ways that have previously been impossible.

In this study, we sampled adult wild salmon returning to the River Etne in 2013 (distinguished from escaped farmed salmon based on morphological characteristics or using DNA and scale samples as detailed below (Madhun et al., 2014; Quintela et al., 2016)), in addition to fish captured upstream of the trap by angling in the same year. Using DNA analysis to individually identify fish captured by angling upstream of the trap, with the data from sampling all fish on the trap, we were able to investigate previously unanswered questions, such as how quickly fish are captured upon entry to the river, the degree to which the angling catch reflects the populations characteristics (i.e., is angling random or selective?), and finally, whether daily upstream migration is linked to biological (i.e., sea age or sex of the fish) or environmental variables such as river discharge and water temperature.

## 2 MATERIALS AND METHODS

### 2.1 The River Etne trap

The trap was installed in the River Etne in 2013 in fishing zone 3 (Figure 1). The holding area is checked daily, and biological measurements (weight and length) are taken from all salmon and sea trout entering the trap. Based upon external morphological characteristics (including body size, fin erosion, coloration), wild salmon and sea trout are thereafter released to continue their upstream migration, while farmed salmon escapees are killed and thereafter sampled to verify their escape status using scale and DNA analysis (Lund & Hansen, 1991; Madhun et al., 2014; Quintela et al., 2016). Fish were identified as either farmed or wild; hybrids were not identified. A small part of the adipose fin of all wild and farmed fish entering the trap is clipped and stored in ethanol for later genotyping. Scale samples are also taken from all fish to perform age analysis of the fish and verify their wild vs. farm status. Thus, the trap and the described sampling regime allow for biological and genetic data to be taken from every salmonid entering the River Etne.

In 2013, the trap was operated from week 20 (May 13) to week 46 (November 17). Hydrographical data pertaining to river discharge and water temperature were accessed through the Norwegian Water Resources and Energy Directorate (NVE: www4.nve.no/en/Water/Hydrology). There were two discharge measurement stations, one where the Etne River meets Stordalsvatnet (59°68′N, 6°01′E) and another in the river branch which comprises fishing zone 27 (Sørelva) (59°65′N, 5°99′E) (Figure 1). During the experimental period (trap period), discharge was higher, on average, at measuring station 1, and the variation in discharge was less at measuring station 2 due to the presence of a hydropower dam on that branch of the river. The two discharges were pooled together for the subsequent analysis as the area where the trap is located would receive a combined flow from both points. The daily number of fish entering the trap during the trap period, the daily number of fish caught by angling during the angling season, daily average water discharge (m/s^3^) and water temperature (°C) are shown in Table 1.

**Table 1:** Daily water temperature and discharge, number of fish recorded each week for the trap, the fishery, and the matched fish, the number of successful anglers (who caught a wild salmon), and the number of females and males recorded each week

Weeks	Daily water temperature (°C)	Daily average water discharge (m/s)	Trap	Fishery	Matched (trap entry)	Matched (caught by anglers)	Number of successful anglers	Female	Male
20	4.94	58.99	2	0	1	0	0	2	0
21	5.73	30.83	11	0	4	0	0	6	5
22	7.88	19.80	23	0	11	0	0	18	5
23	8.50	12.50	16	0	5	0	0	14	2
24	9.94	14.37	23	35	7	17	23	19	4
25	11.11	13.76	81	30	20	13	6	57	24
26	10.70	19.26	129	72	28	25	13	92	35
27	11.10	12.92	70	30	13	10	8	34	35
28	13.01	9.71	31	22	6	8	6	15	16
29	11.77	20.66	276	40	25	20	11	163	112
30	16.83	9.07	83	11	2	9	3	34	49
31	15.81	10.95	92	23	10	29	8	50	42
32	13.94	29.14	37	51	3	2	10	21	16
33	13.42	23.68	26	2	0	0	0	8	18
34	12.79	22.31	27	0	0	0	0	7	20
35	13.25	9.63	71	0	0	0	0	27	44
36	11.82	25.66	61	0	0	0	0	29	32
37	12.35	14.92	12	0	0	0	0	7	5
38	10.57	21.91	22	0	0	0	0	6	16
39	9.53	17.39	11	0	0	0	0	3	8
40	8.23	10.25	3	0	0	0	0	0	3
41	8.29	24.68	9	0	0	0	0	3	6
42	7.61	12.16	9	0	0	0	0	2	5
43	6.93	32.89	1	0	0	0	0	0	1
44	6.39	49.70	7	0	0	0	0	1	6
45	5.64	28.46	5	0	0	0	0	3	2
46	4.78	31.29	2	0	0	0	0	1	1
Total	**–**	**–**	**1,140**	**316**	**135**	**133** a	**90**	**622**	**512**

Daily water discharge for the two measuring stations was pooled for the analysis. For the matched data, the week in which fish entered the trap and the week in which fish were caught are shown.

Two matched fish had missing data for their week of capture. The angling period comprised weeks 24–33.

### 2.2 The River Etne rod fishery (angling)

In 2013, the rod fishery lasted from week 24 (June 15) to week 33 (August 15). All rod catches must be reported to the fishery managers, and anglers were also requested to submit a scale sample and biological measurements to the Institute for Marine Research (IMR), Bergen, for DNA and age analysis. This then allows for the genetic matching of fish between the trap and the rod fishery. In 2013, of the 396 salmonid fish caught during the fishing season (http://etnelaks.no/Fangstar/Laksebors%202013.html), 215 scale samples were given to IMR by anglers. Data relating to angling effort (time each angler spent fishing per day or the total number of anglers per day) were not available, as fishing tickets for this particular river are sold in advance.

### 2.3 Genotyping

All salmon sampled in the trap and captured by angling were initially genotyped using 18 microsatellite markers. DNA extraction and amplification of the 18 microsatellite markers were performed as described in Quintela et al. (2016), with the addition of genetic sex markers to one of the multiplexes: Exon 2 and Exon 4 (modified from Eisbrenner et al., 2014). An additional set of 13 microsatellite markers (total of 31 markers for all fish) were amplified in two multiplexes (MP1: Ssa405, Ssa412 (Cairney, Taggart, & Hoyheim, 2000), Ssa98 (O'Reilly, Hamilton, McConnell, & Wright, 1996), SsOSL25 (Slettan, Olsaker, & Lie, 1995), SSsp2215 (Paterson, Piertney, Knox, Gilbey, & Verspoor, 2004), EST107, EST68 (Vasemagi, Nilsson, & Primmer, 2005) and MP2: EST28, EST19 (Vasemagi et al., 2005). Ssa407 (Cairney et al., 2000), Ssleer15.1 (U86708), Sleen82 (U86706), and Sleel53 (U86704)). Both PCRs were performed in a 7.8 μl reaction volume, which consisted of 0.8 μl of extracted DNA elute, 0.5× KAPA2GTM Fast HotStart ready mix (2**−**) from KAPABIOSYSTEMS (www.kapabiosystems.com), and varying concentrations of primers (details available upon request). Reactions were carried out on a ABI9700 thermocycler and consisted of an initial denaturation step of 150 s at 94°C, followed by nine cycles of denaturation at 95°C for 25 s, annealing at 58°C for 30 s, and extension at 72°C for 25 s; then followed by 28 cycles of denaturation at 95°C for 25 s, annealing at 53°C for 30 s, and extension at 72°C for 25 s; and finally, an extension step of 10 min at 72°C. The PCR products from the two multiplexes were physically mixed before fragment analysis on an ABI 3730XL Genetic Analyser and sized by a 500LIZ™ size standard. Size estimation and scoring of alleles were conducted in GENEMAPPER 5.0, by two persons who evaluated the results independently. Individual salmon captured by angling were matched back to a trap individual (i.e., themselves) using CERVUS version 3.0 (Kalinowski, Taper, & Marshall, 2007). The maximum allowed loci mismatch was set to five (of 31 markers). The highest number of mismatched loci of the matched individuals was 4, and the average *p* value for the likelihood of identity match was 5.99e‐34.

### 2.4 The data and data sets used for analysis

The raw data consisted of (1) the biological measurements, tissue, and scale samples taken at the trap for each individual fish which entered the trap during the trap period, (2) the biological measurements and scale samples provided to IMR by the anglers during the angling season, and (3) the biological measurements (no tissue/scale samples) recorded for each individual caught during the angling season (accessed through www.etneelva.no). The weekly numbers of fish entering the trap and caught by angling are presented in Table 1.

As scale samples were not supplied for all salmon captured by angling in 2013, the aforementioned data were divided into several partially overlapping datasets in order to address the various questions. In all these sets, all farmed salmon were removed prior to analyses. To assess whether angling is selectively harvesting certain body sizes of the river population, the first dataset consisted of (1) all salmon which entered the river daily through the trap from week 20 until week 46 (entire trap period), and a truncated dataset including (2) all salmon which entered the river up until the end of the fishing season (week 33). The datasets consisted of individual data on day of trap entry (day of the year), fish size, sea age and sex, and whether the fish had been matched using DNA analysis to an angling sample that was rod‐caught and killed above zone 3.

To assess whether daily numbers of salmon entering the trap was influenced by day of the year, daily river discharge, daily water temperature, sea age, or sex of the fish, the second dataset consisted of number of fish entering the trap each day throughout the entire trap period (from week 20 until week 46).

To investigate what influenced the probability of being caught by angling after entering the river, the final dataset consisted of only the individuals that entered the river during the angling season (week 24 to week 33). Fish entering the trap before the angling season would have biased our calculations, while those entering after would not have been eligible for rod capture; therefore, these individuals were removed prior to analysis.

### 2.5 Statistical analysis

Analysis was carried out using the free statistical software R version 3.3.1 (R Core Team, 2016).

#### 2.5.1 Comparison between the trap population and the fish captured by angling

To investigate whether angling was size selective on the entire river population, a general additive model (GAM) was fitted using the *gam* function from the *mgcv* package in R (Wood, 2017). The response variable, logged and centered weight in grams, was a continuous variable. The explanatory variables were a smooth spline of day of the year (i.e., day of entry to trap) with subclasses for sea age and sex: a factor variable that consisted of eight levels for each unique combination of sea age (1–4 years) and sex (male and female), and rod capture (binary factor: 0—not caught, 1—caught), and their two‐way parametric interaction:

(1) $$\begin{array}{cc} \text{Size}∼s(\text{day})+ & s(\text{day},\text{sea age}\&\text{sex})+\\ & +s\left(\text{day},\;\text{rod‐capture}\right)+\text{rod}\;{\text{capture}}^{*}\text{sea age}\&\text{sex} \end{array}$$

Where the s indicates a smoothing function. The rod‐captured individuals in the above model were the matched individuals only, as it was not possible to unequivocally match the remaining rod‐caught salmon back to the trap.

#### 2.5.2 Timing and triggers of river entry

A GAM was used to investigate whether daily numbers of fish migrating into the trap was influenced by day of the year, daily river discharge, daily water temperature, and sea age or sex of the fish. The response variable was the number of fish entering the trap each day throughout the trap period and was modeled using a negative binomial distribution with a log‐link function. As above, sea age and sex were combined into a factor variable that consisted of eight levels for each unique combination of sea age (1–4 years) and sex (male and female). Day of the year, daily discharge, and daily water temperature were modeled as smooth splines, with subclasses of sea age and sex for day of the year. Degrees of freedom for the smoothers were set to 3 to ensure against overfitting (Wood, 2006). Daily numbers of each sea age and sex were initially included as parametric variables:

(2) Number∼s(discharge)+s(temperature)+s(day,age&sex)+age&sex

#### 2.5.3 Timing of capture by angling

To investigate the probability of rod capture after river entry, a GAM was fitted using the *gam* function in *mgcv* using a binomial distribution. The response variable was binary and consisted of assigning individuals either a 1 (captured by rod angling and genetically matched back to a trap individual, including individuals that were caught and then released) or 0 (presumed not captured by rod angling). Not all fish captured by angling were subsequently submitted as scale samples to IMR; therefore, it is possible that some of the fish assigned a 0 were in fact caught; however, it was not possible to establish this with the present data. Data exploration revealed that fish size was correlated to sea age; therefore, only sea age was included as a covariate in the model. The model covariates included the continuous covariates of exposure to angling risk (day of rod capture—day of trap entry for caught individuals or final day of angling season—day of trap entry for uncaught individuals), with subclasses for the factor covariates of sea age (4 levels) and sex (2 levels), their parametric interaction term, the continuous parametric covariate of daily water temperature, and a smoother for daily water discharge. Exposure period was included as a smooth term:

(3) $$\begin{array}{cc} \text{Caught}∼s(\text{exposure, by sea age})+ & s(\text{exposure, by sex})+s(\text{discharge})\\ & +\text{temperature}+\text{age}*\text{sex} \end{array}$$

The fit of all of the above models were assessed using the *gam.check* function in the *mgcv* package and plots of the Pearson residuals against fitted values and model covariates. The *anova* function was used for each of the above models to assess the significance of the parametric and smooth model terms.

In the model investigating the factors influencing timing of river entry, the diagnostic plots revealed one extreme residual, which was removed and the analysis was repeated without the outlier. Although there were some slight differences in the model results between the initial and outlier‐free model, Akaike information criterion (AIC) values differed by <2; therefore, the initial model was used in all subsequent analyses.

### 2.6 Ethics statement

All welfare and use of experimental animals were performed in strict accordance with the Norwegian Animal Welfare Act. In addition, all personnel involved in this experiment had undergone training approved by the Norwegian Food Safety Authority (FDU permit number 34273‐1), which is mandatory for all personnel handling fish.

## 3 RESULTS

### 3.1 Summary of the data

A total of 1,143 wild Atlantic salmon were sampled on the fish trap in 2013, and thereafter genotyped. Three of these individuals were removed prior to any analysis due to missing biological data, six individuals could not be genetically sexed, and 24 individuals had no data on their sea age. This left a total of 1110 individuals for statistical analysis. The trap operated from week 20 to week 46, while the angling season was open from week 24 to 33. Of the 1,110 salmon analyzed, 225 entered the river after the angling season had closed and were therefore ineligible to be captured.

Of the 396 salmonids rod‐caught during the fishing season, 316 were found to be wild Atlantic salmon (others were sea trout). Of these 316 wild Atlantic salmon, 66 salmon were rod‐caught below the trap in zones one and two (see Figure 1); therefore, 250 wild salmon individuals were rod‐caught upstream of the trap during the angling season. Of the total 316 wild salmon caught by angling in all zones, 43 were released alive.

Of the 215 scale samples provided by anglers to IMR (i.e., of the 316 total Atlantic salmon catch in all zones), 160 fish were rod‐caught above the trap and thus eligible to be matched back to a trap individual, of which 135 fish were successfully matched back to the trap as wild Atlantic salmon. The remaining 25 fish (of a total 215 samples provided by anglers) that were rod‐caught above the trap and which could not be matched had presumably jumped the trap. Two individuals that were rod‐caught above the trap were released after rod capture.

#### 3.1.1 Comparison between the trap population and the fish captured by angling

Of the total 1110 salmon statistically analyzed, 133 (two individuals were removed due to lack of biological data) salmon were successfully genetically identified in the reported samples given by anglers to IMR. Two individuals that were caught and released were designated as 0 (not rod‐captured) as these individuals were not technically removed from the river population by angling, leaving 131 individuals designated as rod‐caught. Of the total of 1110 salmon entering the River Etne in 2013, 885 of them ascended in the period prior to the fishing season and up to the end of the fishing season (i.e., were exposed to potential angling pressure).

**Figure 2:**
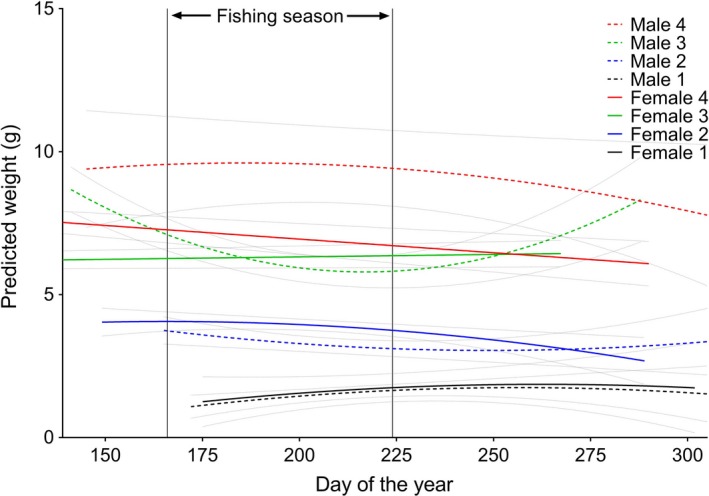
Predicted smooths of size (weight, g) of each sea age and sex class over time. The fishing season is indicated between the vertical lines. 95% confidence intervals are shown by the stippled lines

For the entire river population, weight increased as sea age increased within each sex: males and females of sea age 1 were the smallest individuals while males of sea age 4 were the largest on average (Table 2, Figure 2). The estimated smoothers of day of the year, and subclasses of female sea age 1 and 2, and male sea age 1, 2, and 3 within day of the year smoothers were significantly nonlinearly associated with weight (Table 2). Thus, the size distribution of the fish changed over time dependent upon their sex and sea age, with older fish displaying larger sizes earlier than younger fish, apart from sea age 2 and 3 males where size decreased and increased again over time (Figure 2).

**Table 2:** ANOVA output from the general additive model investigating the relationship between body size and day of the year of trap entry, sex and sea age, and whether a fish was caught by angling or not for all trap individuals

Parametric terms	*df*		*F* value	*p* value
Caught (factor, 2 levels)	1		1.78	.18
**Sea age & sex (factor, 8 levels)**	**7**		**191.40**	**<2e−16**
Caught: Sea age & sex interaction	7		1.27	.26

Approximate smooth terms	Estimated *df*	Ref. *df*	*F* value	*p* value
s(Day)	0.97	0.97	1.19	.28
**s(Day, by female 1)**	**2.39**	**2.69**	**4.37**	**.01**
**s(Day, by female 2)**	**1.32**	**1.62**	**7.71**	**.01**
**s(Day, by female 3)**	**0.88**	**0.88**	**0.57**	**.48**
s(Day, by female 4)	0.88	0.88	3.53	.08
**s(Day, by male 1**	**2.12**	**2.49**	**8.72**	**.00**
**s(Day, by male 2)**	**1.57**	**1.95**	**3.17**	**.03**
**s(Day, by male 3)**	**2.41**	**2.72**	**3.88**	**.04**
s(Day, by male 4)	1.59	1.80	1.69	.27
s(Day, by not caught)	0.51	0.51	0.02	.92
s(Day, by caught)	0.51	0.51	0.06	.86

Significant variables are shown in bold. *df*, degrees of freedom. Females and males numbered 1 to 4 indicated sea ages 1 to 4 within each sex.

**Figure 3:**
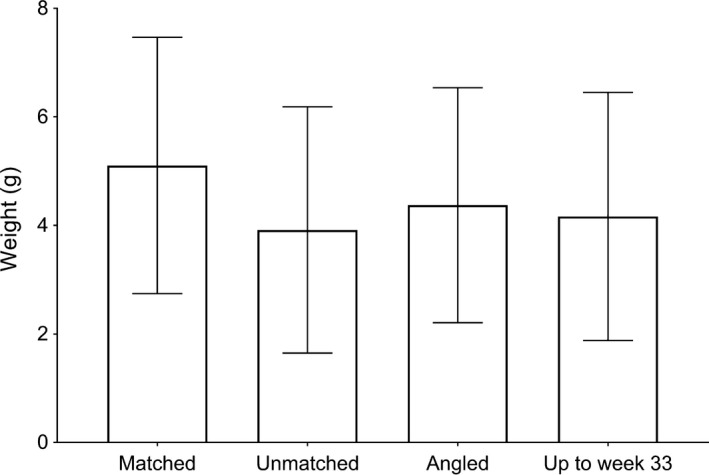
Average weight (g) and standard deviation for all rod‐caught and killed fish that were matched back to the trap, those fish that entered the trap that were not matched back to an angled fish over the entire trap period, all fish rod‐caught and killed during the fishing season, and all fish that entered the trap until week 33 (end of the fishing season) that were not matched back to an angled fish

The age‐based size distribution of the salmon captured by angling and matched back to the trap with DNA (*N* = 133) was not significantly different to the aged‐based size distribution of the population as estimated from the trap data, neither alone nor as an interaction with sea age and sex or as a subclass of the smooth of day of the year (Table 2, Figure 3). The body size distribution of all the salmon captured by angling (*n* = 316), sorted into the size categories (because not all rod‐captured salmon were aged) small (0–3 kg), medium (3–7 kg) and large (7+ kg) weight classes, was similar to the body size distribution of the individuals ascending the trap (small: 33% and 38% (*p* = 0.11), medium: 56% and 51% (*p* = 0.11), and large 11% and 11% (*p* = 1), for the angled and trap individuals, respectively).

#### 3.1.2 Timing and triggers of river entry

**Figure 4:**
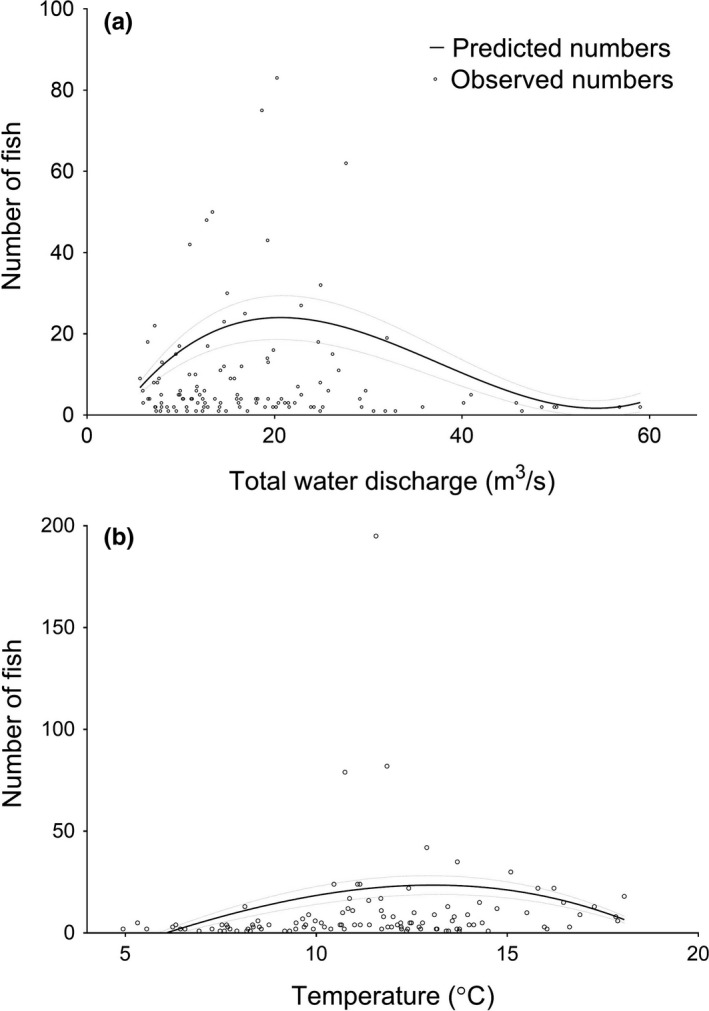
Predicted smooths of the number of fish entering the trap per day for (a) the range of total water discharge (m^3^/s) and (b) water temperature (°C). 95% confidence intervals are shown as stippled lines

Water temperature ranged between 4.9 and 18.1°C, and total discharge was between 14.2 and 59 m^3^/s over the entire period in which the trap was in operation. The relationship between water discharge and daily number of fish entering the trap was significant. The highest number of fish entering the trap occurred when the water discharge was ~20 m^3^/s (Table 3, Figure 4a). Similarly, there was a significant nonlinear relationship between the number of fish entering the trap each day and the daily water temperature (Table 3, Figure 4b), and the peak in the number of fish entering the trap occurred when the water temperature was ~11°C.

**Table 3:** ANOVA output from the general additive model investigating the relationship between number of fish entering the trap daily and day of the year, sex and sea age, water temperature, and discharge

Parametric terms	*df*		Chi square value	*p* value
**Sea age & sex (factor, 8 levels)**	**7**		**16.99**	**0.02**

Approximate smooth terms	Estimated *df*	Ref. *df*	Chi square value	*p* value
**s(Discharge)**	**2.96**	**3.00**	**134.76**	**2.0e−16**
**s(Temp)**	**2.86**	**2.98**	**36.10**	**1.3e−07**
**s(Day, by female 1)**	1.00	1.00	3.65	0.06
**s(Day, by female 2)**	**2.86**	**2.98**	**77.40**	**2.0e−16**
**s(Day, by female 3)**	**2.69**	**2.91**	**57.17**	**2.9e−12**
s(Day, by female 4)	**2.52**	**2.84**	**14.15**	**2.7e−03**
**s(Day, by male 1**	**2.78**	**2.96**	**56.86**	**9.7e−11**
**s(Day, by male 2)**	**2.83**	**2.98**	**56.17**	**1.4e−11**
**s(Day, by male 3)**	**2.67**	**2.92**	**24.00**	**2.0e−05**
s(Day, by male 4)	1.89	2.12	4.93	0.11

Significant variables are shown in bold. *df*; degrees of freedom. Females and males numbered 1–4 indicated sea ages 1–4 within each sex.

**Figure 5:**
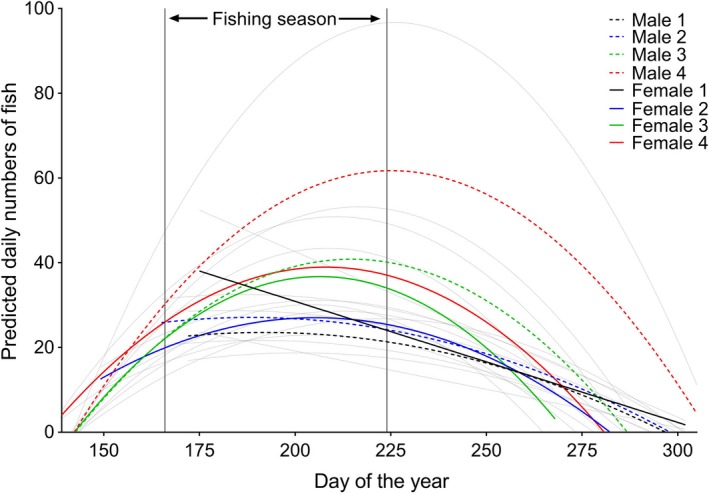
Predicted number of fish entering the trap per day for each sea age and sex class. The fishing season is indicated between the vertical lines. 95% confidence intervals are shown by the stippled lines

**Figure 6:**
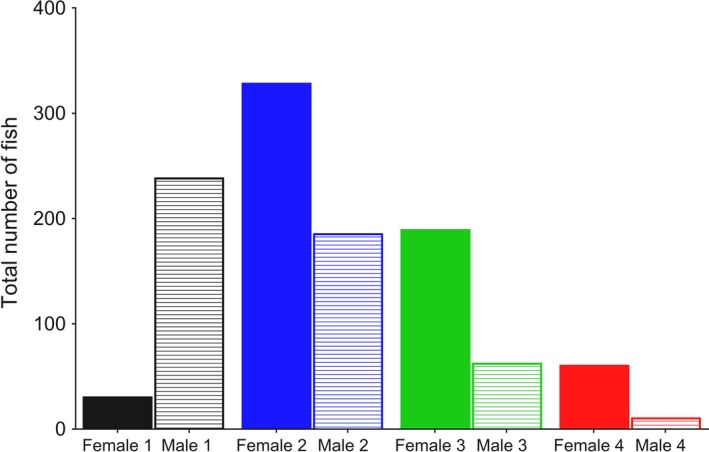
The total number of male and female salmon from each sea age class observed throughout the trap period

A significant nonlinear relationship was observed between the number of fish entering the trap and day of the year, conditional on the sea age, and sex combination (Table 3, Figure 5). The number of sea age 2, 3, and 4 female and males, and sea age 1 males, increased nonlinearly over time before reaching a peak and decreasing, while the number of sea age 1 females exhibited a linear relationship over time (Figure 5). Older individuals entered the trap earlier than younger individuals, and on average, sea age 2 and 4 females entered before males, while sea age 1 males entered before females (Figure 5). The overall sex ratio (female: male) of the river population was 1.2:1 (Table 1). However, this ratio was also dependent upon age: There were higher numbers of sea age 1 males than females, and higher numbers of sea age 2, 3, and 4 females than males (Figure 6).

#### 3.1.3 Timing of capture by angling

**Figure 7:**
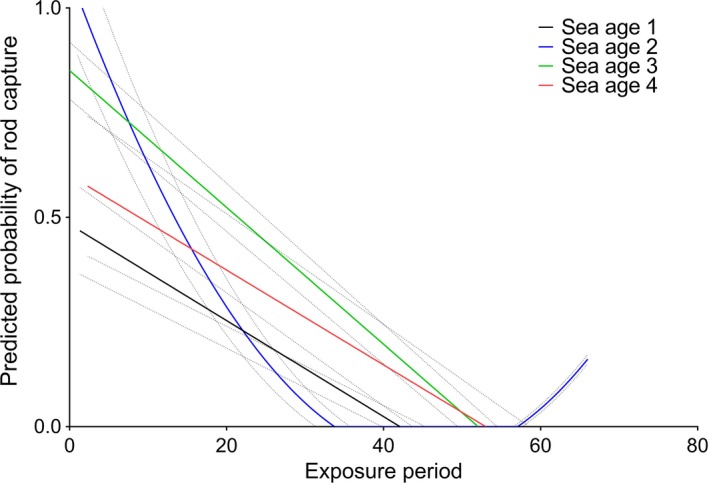
The predicted probability of rod capture of each sea age over time within the fishing season, where exposure period is the time between the end of the fishing season and the day of entry to the trap

During the angling season, 824 individuals ascended the river. Of these, 18 had missing biological data (either age or sex information) and these individuals were removed prior to analysis, leaving 806 individuals with biological data. Of the total of 160 salmon captured above the trap by angling, 114 could be matched genetically back to an individual that entered the trap during the angling season (i.e., one of the 806 individuals) using their given scale sample. Based upon these 114 individuals, the average period between trap entry and rod capture was 11 days. The estimated smoother of exposure period with subclasses for sea age was significant, with the probability of rod capture decreasing as time in the river (i.e., exposure period) increased in general and older fish having a higher probability of rod capture sooner than younger fish, apart from sea age 2 individuals (Table 4, Figure 7). Temperature and discharge were negatively associated with probability of capture (Table 4). The sex of the fish was not significantly associated with the probability of rod capture (Table 4).

**Table 4:** ANOVA output from the general additive model investigating the probability of rod capture and exposure time, sea age and sex, discharge, and temperature for caught and uncaught individuals during the fishing season

Parametric terms	*df*		Chi square value	*p* value
Sex (factor, 2 levels)	1		1.43	.23
Sea age (factor, 4 levels)	3		0.46	.93
**Temperature**	**1**		**40.39**	**2.08e−10**

Approximate smooth terms	Estimated *df*	Ref. *df*	Chi square value	*p* value
**s(Exposure period, by sea age 1)**	**1.00**	**1.00**	**26.22**	**.00**
s(Exposure period, by sea age 2)	4.89	5.03	10.08	.07
**s(Exposure period, by sea age 3)**	**1.00**	**1.00**	**14.93**	**.00**
**s(Exposure period, by sea age 4)**	**1.00**	**1.00**	**11.05**	**.00**
s(Exposure period, by females)	7.34	7.88	16.12	.05
s(Exposure period, by males)	0.04	0.08	0.01	.94
**s(Discharge)**	**5.73**	**6.79**	**20.75**	**.00**

Significant variables are shown in bold. *df*, degrees of freedom.

## 4 DISCUSSION

The upstream migration trap located in the River Etne, which permits the vast majority of salmon entering this major river system to be sampled daily, is the first of its kind in northern Europe. When data from the trap was combined with the angling catch and individual DNA profiling, we were able to connect individual fish's time of entry into the river and subsequent capture by angling. This system has thus provided several unique insights into the potential components underpinning angling‐induced selection in a salmon population. The main results of this study can be summarized as follows: (1) There was no significant difference in weight observed between the salmon captured by angling and matched to the trap using DNA analysis, and the population itself, indicating random size harvest in the rod fishery. (2) Larger, older fish tend to enter the river earlier, and there is an inverse sex bias of sea age, where older females are more abundant than males and vice versa. (3) The relationship between river discharge, water temperature, and number of salmon entering the river indicates there are potential thresholds of water discharge and temperature for river entry. (4) The probability of being captured by angling decreased with the numbers of days after which the fish had entered the river. That is, fish are more likely to be captured quickly after river entry, with a mean exposure period of 11 days. Collectively, these data demonstrate that at least in this river system, salmon harvest via angling is random in relation to the size profile of fish entering the river system during the fishing season. Put alternatively, anglers more or less randomly catch the fish ascending the river at the time of angling. Therefore, as there is a major difference in the demography of fish (age/size/sex) entering the river during the migration period, we conclude that the timing of the fishing season is most likely to represent the primary driver of any size or sex‐specific selection potentially arising from angling.

### 4.1 Comparison between the trap population and the fish captured by angling

Atlantic salmon populations display a great diversity in a wide range of phenotypic traits. Many of these traits display an underlying genetic basis and potentially represent adaptations to local conditions (Garcia de Leaniz et al., 2007; Taylor, 1991). Traits that are of potential significance in the context of fisheries exploitation, namely, timing of return to freshwater (Quinn, McGinnity, & Reed, 2016; Stewart, Smith, & Youngson, 2002), and size/age of maturation (Ayllon et al., 2015; Barson et al., 2015), both vary within and among populations, and display an underlying genetic basis. Consequently, selectively harvesting salmon populations could potentially elicit a heritable response in these traits if the anglers target a specific life history or size is targeted over others.

Although fisheries‐induced evolution is a contentious issue (Dunlop et al., 2009), commercial fishing has affected size at age in certain fish stocks (Heino & Godoe, 2002; Jørgensen et al., 2007), and several studies suggest that angling exerts a size‐selective pressure on fish populations (Hard et al., 2008; Lewin, Arlinghaus, & Mehner, 2006). For example, angling exploitation was believed to have contributed toward a shift in decreasing size of Atlantic salmon in Irish rivers (Quinn et al., 2006). Similar trends of decreasing body size over time have been observed for salmonids in rivers in Spain and North America and have also been linked to selection from anglers (Saura et al., 2010) and commercial fisheries (Kendall et al., 2013). In contrast to the above, our results from the River Etne indicate that the anglers randomly harvest fish that ascend the river at the time of fishing (Table 2). Although the analysis was conducted on a subset of the angling data, we found that body size did not differ significantly between the rod‐captured and killed salmon and the fish entering the trap based on body size categories. Levels of angling mortality will depend on various factors, including angling effort, which was not measured in the present study, and specific river regulations, such as the duration and timing of the angling season (Lewin et al., 2006). While angling mortality alone may not be selectively targeting for body size, the timing and duration of the angling season may thus be highly influential drivers of angling selection.

### 4.2 Timing and triggers of river entry

Size/age and time of river entry is inherently linked in salmonids: larger, older salmon tend to enter rivers earlier than younger, smaller salmon (Borgstrøm et al., 2010; Davidsen et al., 2013; Gurney, Bacon, Malcolm, Maclean, & Youngson, 2015; Thorstad et al., 2011; Webb et al., 2007). In the present study, we also found that larger, older fish entered the river earlier than smaller, younger fish (Table 3, Figures 2, 4). Sea ages 2 to 4 tended to enter the river before the fishing season, while individuals aged 1 year tended to enter after the fishing season (Figures 2, 4). Our data also found that females within certain age classes enter the river earlier than males, and that older females were more abundant than older males, while there were more males of sea age 1 than females (Figures 5, 6). Earlier river entry of female salmon has been found in other studies (Dahl et al., 2004; Pérez, Izquierdo, de la Hoz, & Garcia‐Vazquez, 2005; Saura et al., 2010), and it has also been suggested that angling may cause a selection bias toward one sex (Kendall & Quinn, 2013). Therefore, the placement of the angling season, in relation to when the different sexes or age classes of fish enter the river, will influence the selectiveness of the catch. In this context, Quinn et al. (2006) investigated long‐term data of size and run times of Atlantic salmon in three Irish fisheries. They found a shift from early to later migration in all rivers and suggested that this may have been caused by selection resulting from angling exploitation (Quinn et al., 2006). Similarly, Consuegra et al. (2005) demonstrated that long‐term selective pressure from anglers was causing differential mortality of genetically distinct early‐running Atlantic salmon populations on the Iberian coast. Changes in the run times of Chinook and sockeye salmon from past to present may also have been caused by the selective exploitation by commercial fisheries of the earlier‐returning, larger salmon (Quinn et al., 2016).

While there is a genetic component to timing of migration (Hansen & Jonsson, 1991; Stewart et al., 2002), environmental variables such as river discharge and water temperature may also affect migration (Dahl et al., 2004; Quinn et al., 2016). Temperature is an important factor for many developmental processes in salmonids and serves as an environmental cue for initiating migration, while water flow can influence the accessibility of rivers for migrating salmon (Jonsson & Jonsson, 2009). Studies have generally found that fish will migrate upstream when river water discharge is increasing and between optimum water temperature levels (Jonsson & Jonsson, 2009; Thorstad et al., 2008).

Our results indicate that both water temperature and discharge positively influence migration up river within certain thresholds. The present study found the numbers of fish entering the river peaked around 11°C; similarly, there was a peak in numbers entering the river when the discharge was around 20 m^3^/s (Table 1, Figure 4a,b). Jonsson, Jonsson, and Hansen (2007) found both temperature and water flow to be positively associated with migration within certain thresholds and in certain months of the year in the River Imsa in southwest Norway. They observed the highest numbers of salmon ascending the river when water temperature was between 10 and 12.5°C and discharge was between 12.5 and 15 m^3^/s (Jonsson et al., 2007). L'Abeé‐lund and Aspås (1999) suggest threshold values for angler's catch of 250 m^3^/s for river discharge and 8°C for water temperature in the River Guala in Norway, while Gee (1980) found no catches above 16°C in the River Wye. Thorstad, Heggberget, and Økland (1998) found that increasing water discharge positively influences migration into rivers for wild salmon.

The effects of river discharge and water temperature are complex as they are often interrelated (Thorstad et al., 2008) and will vary between rivers and populations. It has been suggested that water flow may not be as important for migration in larger rivers as in smaller rivers (Davidsen et al., 2013; Jonsson & Jonsson, 2009). A study investigating the yearly variation in angling catch of grilse in Norwegian rivers found that the association between catch and water discharge decreased when hydroelectric dams were present in the water course (Otero et al., 2011). Finally, larger fish may be more dependent on water flow than smaller fish, especially in smaller rivers (Jonsson et al., 2007; Karppinen, Erkinaro, Niemela, Moen, & Økland, 2004). Water temperature may be important for migration when there are obstacles in the river, such as ladders or waterfalls, as swimming capability may be impaired outside of optimum temperatures (Richard, Bernatchez, Valiquette, & Dionne, 2014; Thorstad et al., 2008).

### 4.3 Timing of capture by angling

The results of the present study clearly demonstrate that salmon were more likely to be caught sooner after entering the river than later, dependent on sea age (Table 4, Figure 7). The potential evolutionary implications of this observation are significant, and clearly demonstrate that both the timing and duration of the angling season can exert a selective pressure on the population. For example, fish which enter rivers after the angling season has stopped will not be exploited, and fish that enter rivers early in the year (so‐called “springers” in some regions) may be less susceptible to angling pressure if the season is placed so that they have been in the river for some time before angling is permitted. Thorley, Youngson, and Laughton (2007) investigated the exploitation level of different run‐timing groups in the River Spey in Scotland. They observed higher angling mortality, thus implying a higher selective pressure, in early‐running multi‐sea‐winter (MSW) salmon compared to late‐running salmon (Thorley et al., 2007). Similarly, in a temporal study of the River Utsjoki in Finland, Borgstrøm et al. (2010) observed higher angling mortality of MSW Atlantic salmon, which return to the river earlier than smaller one‐sea‐winter (1SW) salmon, leading the authors to suggest a later opening of the fishing season. Our results indicate that older, larger fish are entering the river earlier and before the fishing season, while younger, smaller fish are entering the river after the fishing season has begun. Although we found no evidence for size‐selective harvesting by angling, the differences in run time between the age classes and our findings of higher probability of capture in a shorter exposure period for younger fish indicate that angling may select for certain sea ages within the population. Clearly, timing of the season is the vital factor with respect to the selective nature of angling for the demographic parameters measured in the present study.

The migrating behavior of salmon as they make their way up river to preferred spawning sites will also influence their probability of capture. The upriver migration of salmon has been shown to consist of three distinct phases before spawning: movement up the river, searching near the spawning site, and holding near the spawning site (Økland et al., 2001; Finstad, Økland, Thorstad, & Heggberget, 2005; Thorstad et al., 2011; although see Richard et al., 2014). Thus, fish are more active during the first phase of migration and may be easier to catch during these phases of active movement. Physical barriers that impede the process of migration, such as waterfalls or dams, can cause fish to accumulate and will influence the probability of capture (Karppinen et al., 2004). Thus, the probability of capture is probably dependent on several factors, including migration behavior, river characteristics, including river length, and the length of the angling season and may vary from river to river.

The model found that both water temperature and discharge were negatively associated with probability of capture (Table 4). The temperature and discharge windows during the angling period were narrower than during the entire trap period, and the highest angling catches were recorded below 16°C and 20 m3/s (Table 1), indicating optimal rod‐catch thresholds for this river system, observed in other rivers (L'Abeé‐lund & Aspås, 1999). Several studies have suggested that handling of salmonids may negatively affect their subsequent migration behavior or probability of subsequent capture (Bernard, Hasbrouck, & Fleischman, 1999; Bromaghin, Underwood, & Hander, 2007; Underwood, Bromaghin, & Klosiewski, 2004). That some of the salmon in the present study were re‐captured quickly after being “sampled” in the trap (e.g., one individual was rod‐captured on the same day as they entered the river) suggests that salmon tolerate this type of handling quite well.

## 5 CONCLUSIONS

Our data suggest that certain phenotypes (e.g., fish of different sea ages) will be more at risk of angling pressure at certain times, that is, soon after river entry and within certain water flow and temperature thresholds. Our results indicate that angling mortality is not exerting a size‐selective pressure on salmon, at least in this system. We found that older, larger salmon entered the river earlier than younger, smaller salmon, while younger salmon had a higher probability of rod capture sooner after river entry than older fish, indicating that the placement and duration of the angling season is likely to be the main driver of selective exploitation, and thus fisheries‐induced evolution, of certain size or age classes within the population. As our data and other studies demonstrate, the relationships between angling selection and the traits which may be under selection (migration timing, size, maturation, and sex) are complex and will differ depending on the duration and timing of the angling season and river in question. Similarly, there is spatial and temporal variation in environmental factors among river systems, such as water temperature and discharge, which also influence time of river entry and age at maturity. The use of a resource like the Etne trap, which allows for almost complete sampling of a river population, will allow managers and conservationists to be able to more accurately monitor and predict the effects of fisheries selection on wild populations, and allow better estimates of exploitation rates.

## AUTHOR CONTRIBUTIONS

ØS, VW, and KAG conceived the study. ØS designed and organized the RBW trap project, and conducted sampling and data collection. YT and VW conducted the genotyping and assignment. KAG coordinated the study. ACH conducted statistical analyses and produced the first draft of the manuscript. ACH, YT, VW, ØS, and KAG contributed to data interpretation and writing, and all approved the final manuscript.

## AVAILABILITY OF DATA AND MATERIALS

The datasets supporting the results of this article are available in the Dryad repository (to be completed after manuscript is accepted).
